# Joint effects of meteorological factors and PM_2.5_ on age-related macular degeneration: a national cross-sectional study in China

**DOI:** 10.1265/ehpm.22-00237

**Published:** 2023-01-11

**Authors:** Jiayu He, Yuanyuan Liu, Ai Zhang, Qianfeng Liu, Xueli Yang, Naixiu Sun, Baoqun Yao, Fengchao Liang, Xiaochang Yan, Yang Liu, Hongjun Mao, Xi Chen, Nai-jun Tang, Hua Yan

**Affiliations:** 1 Department of Occupational and Environmental Health, School of Public Health, Tianjin Medical University, Tianjin, 300070, China; 2 Tianjin Key Laboratory of Environment, Nutrition, and Public Health, Tianjin Medical University, Tianjin, 300070, China; 3 Department of Ophthalmology, Tianjin Medical University General Hospital, Tianjin, 300052, China; 4 Laboratory of Molecular Ophthalmology, Tianjin Medical University, Tianjin, 300070, China; 5 Tianjin Key Laboratory of Ocular Trauma, Tianjin, 300070, China; 6 Tianjin Key Laboratory of Urban Transport Emission Research, College of Environmental Science and Engineering, Nankai University, Tianjin, 300071, China; 7 School of Public Health and Emergency Management, Southern University of Science and Technology, Shenzhen, 518055, China; 8 National School of Development, Peking University, Beijing, 100871, China; 9 Gangarosa Department of Environmental Health, Rollins School of Public Health, Emory University, Atlanta, GA, 30322, USA.

**Keywords:** Meteorological factors, Air pollution, Age-related macular degeneration, PM_2.5_, Synergistic effect

## Abstract

**Background:**

Weather conditions are a possible contributing factor to age-related macular degeneration (AMD), a leading cause of irreversible loss of vision. The present study evaluated the joint effects of meteorological factors and fine particulate matter (PM_2.5_) on AMD.

**Methods:**

Data was extracted from a national cross-sectional survey conducted across 10 provinces in rural China. A total of 36,081 participants aged 40 and older were recruited. AMD was diagnosed clinically by slit-lamp ophthalmoscopy, fundus photography, and spectral domain optical coherence tomography (OCT). Meteorological data were calculated by European Centre for Medium-Range Weather Forecasts (ECMWF) reanalysis and were matched to participants’ home addresses by latitude and longitude. Participants’ individual PM_2.5_ exposure concentrations were calculated by a satellite-based model at a 1-km resolution level. Multivariable-adjusted logistic regression models paired with interaction analysis were performed to investigate the joint effects of meteorological factors and PM_2.5_ on AMD.

**Results:**

The prevalence of AMD in the study population was 2.6% (95% CI 2.42–2.76%). The average annual PM_2.5_ level during the study period was 63.1 ± 15.3 µg/m^3^. A significant positive association was detected between AMD and PM_2.5_ level, temperature (T), and relative humidity (RH), in both the independent and the combined effect models. For PM_2.5_, compared with the lowest quartile, the odds ratios (ORs) with 95% confidence intervals (CIs) across increasing quartiles were 0.828 (0.674,1.018), 1.105 (0.799,1.528), and 2.602 (1.516,4.468). Positive associations were observed between AMD and temperature, with ORs (95% CI) of 1.625 (1.059,2.494), 1.619 (1.026,2.553), and 3.276 (1.841,5.830), across increasing quartiles. In the interaction analysis, the estimated relative excess risk due to interaction (RERI) and the attributable proportion (AP) for combined atmospheric pressure and PM_2.5_ was 0.864 (0.586,1.141) and 1.180 (0.768,1.592), respectively, indicating a synergistic effect between PM_2.5_ and atmospheric pressure.

**Conclusions:**

This study is among the first to characterize the coordinated effects of meteorological factors and PM_2.5_ on AMD. The findings warrant further investigation to elucidate the relationship between ambient environment and AMD.

**Supplementary information:**

The online version contains supplementary material available at https://doi.org/10.1265/ehpm.22-00237.

## Graphical Abstract

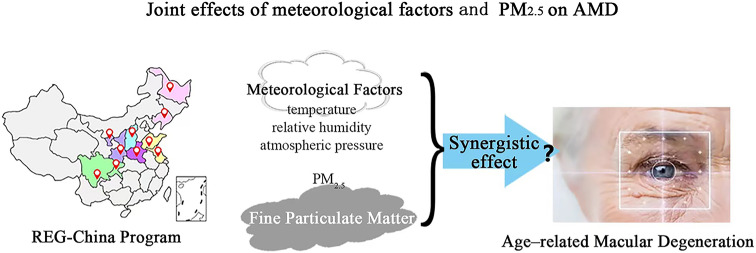

## Introduction

Evidence suggests that meteorological factors affect human health, posing serious risks to the well-being of aging populations in particular [1, 2]. In addition, air pollution is a leading cause of respiratory illness and certain other diseases worldwide [3]. Of special interest regarding meteorological factors and air quality is the increasing global burden of eye disease and concern about its environmental etiology. Previous research in rural China has suggested that long-term exposure to high levels of fine particulate matter (PM_2.5_) is associated with an elevated risk of diabetic retinopathy and glaucoma [4, 5].

Age-related macular degeneration (AMD) is a complex degenerative disease of the retina associated with photoreceptor atrophy and degeneration of the retinal pigment epithelium and choriocapillaris [6]. AMD is estimated to be the third most common cause of blindness and the fourth most common cause of vision impairment globally [7–10]. In China, the prevalence of AMD ranges from 2.44% to 18.98% in persons ages 45–49 and 85–89, respectively [11]. It is anticipated that China will soon have the greatest number of AMD patients of any country in the world, due to the significant growth of its aging population [12].

Risk factors for AMD include interactions between genetic and environmental factors. Recent studies have suggested that air pollution is a potential risk factor for AMD [13, 14]. According to the UK Biobank study, PM_2.5_ is associated with self-reported AMD in participants ages 40–69 [15]. In the Canadian Longitudinal Study, increased PM_2.5_ levels were associated with visual impairments from AMD in the single pollutant model [16]. In a study in Taiwan, AMD risk increased by 19% for every 10 µg/m^3^ increase in PM_2.5_ [14]. Air pollution has been shown to decrease axoplasmic transport in the optic nerve. PM may cause neuroglial damage and inflammatory responses in the retinal structures. Adverse retinal structural features associated with exposure to PM_2.5_ may lead to the development of AMD [15, 17, 18].

Increases in PM_2.5_ concentrations are attributed to various natural environmental factors. Among such factors, weather conditions are some of the most important [19]. The adverse health effects associated with co-exposure to air pollution and meteorological factors may be more serious than effects associated with either air pollution or weather conditions alone [20]. Nevertheless, the impacts of meteorological factors (atmospheric pressure, temperature, relative humidity, etc.) and PM_2.5_ on AMD have rarely been reported.

Given the limited research on AMD and meteorological factors, and the biological plausibility of an association, the current study aimed to elucidate the joint effects of meteorological factors and PM_2.5_ on AMD in a rural study population in China. The findings could provide additional insight for improving prevention and control strategies for AMD.

## Materials and methods

### Study population

The present study population was based on the Rural Epidemiology for Glaucoma in China (REG-China) (Fig. S1), which included populations from 10 provinces, autonomous regions, and municipalities in rural areas of China. A multistage stratified cluster sampling procedure was used to enroll a nationally representative sample of populations. The details of study design were described elsewhere [4, 5] and in Supplemental Methods.

**Fig. 1:**
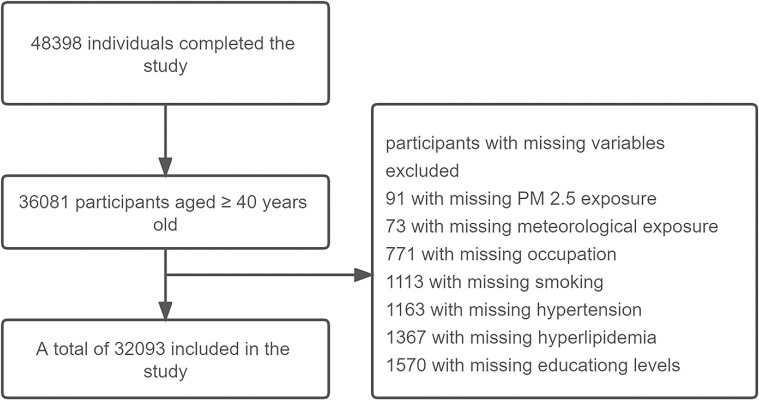
Flow chart of identification of the study population. Interviewers contacted all the selected participants to complete a standardized survey questionnaire and to conduct physical and ocular examinations. In total, 36,081 participants aged ≥40 years old were selected from REG-China study. Among 36,081 participants, 32,093 participants had complete exposure and health data included in the further analysis.

An epidemiologic survey was conducted from June 2017 to October 2018, with recruitment of 52,041 individuals. In this study, 36,081 participants aged ≥40 years old were included in the analysis from the REG-China study. Among 36,081 participants, 32,093 had health data included in the further analysis (Fig. 1). The Tianjin Medical University Research Ethics Committee authorized the research study, and all survey procedures conformed to the Helsinki Declaration Principles. Written informed consent of each subject was obtained prior to the study.

### AMD diagnosis and eye examinations

The definition of AMD in the present study was based on the ICD-9 code (362.51 or 362.52) [21]. AMD was diagnosed clinically by slit-lamp ophthalmoscopy, fundus photography, and spectral domain optical coherence tomography (OCT) [22]. All patients were evaluated by a trained specialist through dilated ophthalmoscopy.

### Exposure assessment for PM_2.5_

An established satellite-based spatiotemporal model was used to estimate PM_2.5_ concentrations at 1-km spatial resolution; detailed procedures have been described elsewhere [4, 5]. As shown in Supplemental Methods, the residential addresses of study participants were geocoded to longitude and latitude. PM_2.5_ exposure levels were matched to participants’ home addresses by latitude and longitude. Average exposure levels of PM_2.5_ from 2007 to 2016 were available and were calculated as long-term exposure levels used in the association analyses.

Briefly, the aerosol optical depth (AOD) product retrieved by the Multi-Angle Implementation of Atmospheric Correction (MAIAC) algorithm was derived from the US National Aeronautics and Space Administration (NASA) Moderate Resolution Imaging Spectrometer (MODIS) satellite. A machine learning algorithm was used to link AOD with other predictors of meteorology, road network, land cover index, and air pollution emissions to estimate PM_2.5_ concentrations. The cross validation showed a high agreement between predicted historical PM_2.5_ concentrations with available ground monitoring data at the annual level (prediction R^2^ = 0.80) [23].

### Exposure assessment for meteorological factors

The ECMWF reanalysis v5 (after this, referred to as ERA5) is the fifth-generation atmospheric reanalysis dataset produced by the Copernicus Climate Change Service at the European Centre for Medium-Range Weather Forecasts (ECMWF).

ERA5 provides hourly estimates of multiple atmospheric, land, and oceanic climate variables (up to 240 variables). The estimates cover the period from January 1950 to the present. The data are available on 137 levels, from the surface up to a height of 80 km, and on regular latitude-longitude grids with a resolution of 0.25 degrees (approximately 28 km). The data was produced using the latest assimilation technology, which combines model data with observations from across the world to produce the best new estimate of the state of the atmosphere [24, 25]. ERA5 data has been proven to have high reliability [26, 27] and therefore is widely used in climate, environmental, and health studies [28, 29].

In this study, ERA5 single-level hourly data was retrieved for 2 meter (m) temperature, 2 m dew temperature, surface pressure, and u and v components of 10 m wind from the Copernicus Climate Data Store utilizing a python script suggested by ECMWF for the 2007–2016 period. After decoding (using the Climate Data Operators tool) and average processing, meteorological element daily mean values of grid points were obtained.

As the relative humidity (RH) is not archived directly in the ERA5, relative humidity is calculated from 2 m temperature and 2 m dew temperature using the following formula: *RH* = *es*(*T_d_*) × 100/*es*(*T*), where es is the saturation vapor pressure at a certain temperature, *es*(*T*) = 6.11 × *e*^(17.67×(^*^T^*^−273.15)/(^*^T^*^−273.15+243.5))^. In addition, in order to match the ERA5 grid points to participants’ home addresses, grid points were interpolated to the study points according to the latitude and longitude of home addresses using a linear interpolate method.

### Statistical analysis

Demographic characteristics were aggregated using the median and quartile range for continuous variables and frequency and percentage for categorical variables. The Mann-Whitney-Wilcoxon test and Fisher’s exact test were used to make statistical comparisons of the demographic characteristics of the various groups. Spearman correlation was used to assess the relationship between PM_2.5_ levels and meteorological factors.

We conducted unconditional logistic regression models to investigate the separate associations of AMD with PM_2.5_ and meteorological factors (temperature, relative humidity, and atmospheric pressure) with adjustment for potential confounding covariates (sex, age, regions, ethnicity, education level, occupation, marital status, personal annual income, smoking status, physical activity time, hypertension, and hyperlipidemia). To examine the joint effects of PM_2.5_ and meteorological factors on AMD, PM_2.5_ levels, temperature, relative humidity, and atmospheric pressure were included in the logistic model simultaneously, adjusting for the same covariates. PM_2.5_ and meteorological factors levels were analyzed as continuous variables and were then categorized into quartiles. The categorization was according to the distribution of PM_2.5_ and meteorological factors, and the lowest quartile group was used as the reference group in the logistic regression model.

To evaluate interaction effects between exposure variables, interactions of PM_2.5_ and meteorological factors were evaluated by using multiplicative and additive interaction terms. The formula of logistics model fit by multiplicative interaction as shown: logit(π) = β_0_ + β_1_X_1_ + β_2_X_2_ + β_3_X_1_X_2_ + γ_1_C_1_ + γ_2_C_2_ + … β_3_ is the multiplicative interaction coefficient. “exp(β_3_) = 1” means no interaction; “exp(β_3_) > 1” represents synergistic effect; “exp(β_3_) < 1” represents antagonism effect [30]. At the additive scale, interaction effects were assessed by three indicators: relative excess risk due to interaction (RERI), attributable proportion (AP), and synergy index (S) [31]. When additive interaction is absent, both RERI and AP are equal to 0 and S is equal to 1. When additive interaction is synergistic effect, RERI or AP will be more than 0, or S will be more than 1 (0 is clearly without the 95% confidence interval (CI) of RERI and AP or 1 is clearly without the 95% CI of S). When additive interaction is antagonism effect, RERI or AP will be less than 0, or S will be less than 1 (0 is clearly without the 95% CI of RERI and AP or 1 is clearly without the 95% CI of S) [32]. PM_2.5_ and meteorological factors were divided into two groups at the 50th percentile (High: >50th percentile and Low: ≤50th percentile). The low level was used as a reference group. Subgroup analyses were used to stratify the association by sex, age, smoking status, and occupation, allowing for assessment of potential modification effects of covariates.

All statistical analyses were performed using IBM SPSS Statistics (version 24.0, IBM Corp, USA) and R Software (version 4.0, R Foundation, Statistical Computing, Vienna, Austria). The two-side P < 0.05 was statistically significant.

## Results

### Description of the study participants

Table 1 shows the baseline characteristics of the study population. Of the 32,093 participants, 833 had AMD, yielding a prevalence rate in the study population of 2.6% (95% CI 2.42–2.76%). Overall, the mean age of participants was 62.4 years, and 39.2% were male. Approximately 63.9% of participants had a lower education level, 90.1% of participants were married, 69.9% of participants were farmers, and 90.4% of participants had an annual personal income of less than 30,000 yuan. Only 24.8% of participants smoked.

**Table 1:** Baseline characteristics of case and control groups.

	**Individuals with AMD**	**Individuals without AMD**	**Total**
Participants, No.	833	31260	32093
Sex, No. (%)			
Male	323 (38.8)	12248 (39.2)	12571 (39.2)
Female	510 (61.2)	19012 (60.8)	19522 (60.8)
Age, mean (SD), year	67.6 (9.4)	62.3 (11.3)	62.4 (11.3)
Education level, No. (%)			
Primary school or less	636 (76.4)	19868 (63.6)	20504 (63.9)
Middle or high school	189 (22.7)	10094 (32.3)	10283 (32.0)
College or more	8 (1.0)	1298 (4.2)	1306 (4.1)
Marital status, No. (%)			
Never married	22 (2.6)	661 (2.1)	683 (2.1)
Married/Common	691 (83.0)	28115 (90.3)	28806 (90.1)
Divorced/Widowed	120 (14.4)	2375 (7.6)	2495 (7.8)
Ethnicity, No. (%)			
Han	751 (90.2)	28330 (90.6)	29081 (90.6)
Hui	76 (9.1)	2008 (6.4)	2084 (6.5)
Others	6 (0.7)	922 (3.0)	928 (2.9)
Occupation, No. (%)			
Farmer	607 (72.9)	21822 (69.8)	22429 (69.9)
Non-farmer	226 (27.1)	9438 (30.2)	9664 (30.1)
Personal annual income, No. (%)			
<30000	781 (93.8)	28232 (90.3)	29013 (90.4)
30000–80000	52 (6.2)	2816 (9.0)	2868 (8.9)
>80000	0 (0)	210 (0.7)	210 (0.7)
Smoking status, No. (%)			
Never	659 (79.1)	23487 (75.1)	24146 (75.2)
Former/Current	174 (20.9)	7773 (24.9)	7947 (24.8)
PM_2.5_ exposure, mean (SD), µg/m^3^	65.2 (15.3)	59.7 (14.3)	63.1 (15.3)
Temperature, mean (SD), °C	13.9 (3.6)	13.2 (4.0)	13.2 (4.0)
Relative humidity, mean (SD), %	70.1 (7.6)	66.8 (7.2)	66.9 (7.2)
Atmospheric pressure, mean (SD), hPa	916.9 (61.5)	950.8 (65.4)	949.9 (65.5)

The average annual PM_2.5_ exposure during the study period was 63.1 ± 15.3 µg/m^3^. PM_2.5_ concentrations were higher in AMD individuals than in the population without AMD, and mean levels of temperature and relative humidity at home addresses of AMD patients were slightly higher than for other individuals. AMD patients also experienced lower atmospheric pressure exposure.

**Fig. 2:**
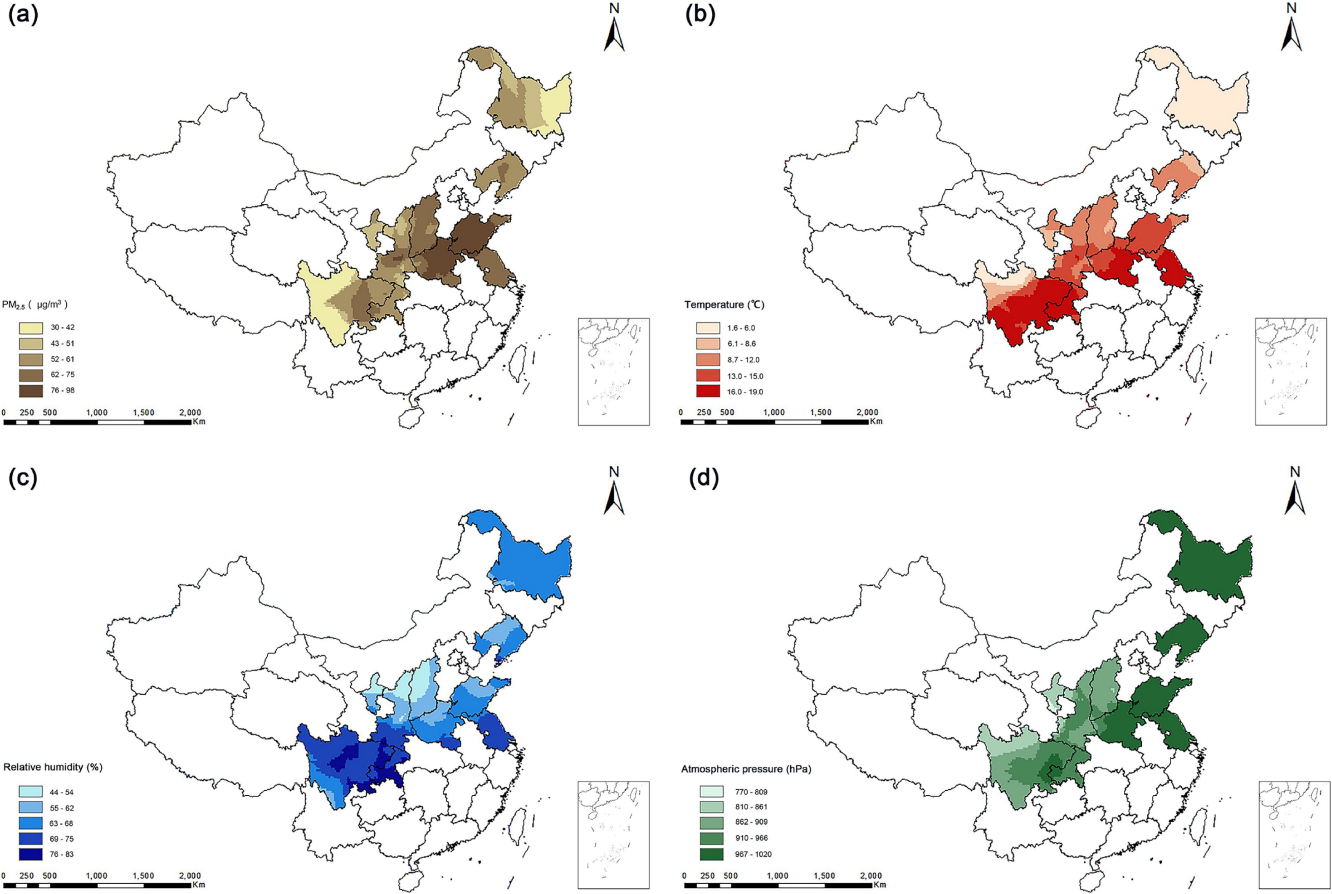
Characteristics of meteorological factors and ambient PM_2.5_ levels in 10 provinces of China. (a): mean levels of PM_2.5_ from 2007–2016 by established satellite-based spatiotemporal models; (b, c, and d): meteorological data (temperature [b], relative humidity [%; c], and atmospheric pressure [hPa; d]) from 2007–2016 were calculated by ERA5. Meteorological data were matched to participants’ home addresses by latitude and longitude as the long-term exposure at the individual level.

As shown in Fig. 2(a), the highest mean PM_2.5_ concentrations were in South Central China (88.2 ± 5.9 µg/m^3^) and the lowest in Northwest China (52.6 ± 12.9 µg/m^3^). The highest mean annual temperature was in Southwest China (16.8 ± 1.4 °C), while the lowest mean annual temperature was in Northeast China (6.0 ± 2.1 °C) (Fig. 2(b)). North China had a lower relative humidity (60.1 ± 1.2%) compared to Southwest China (76.5 ± 3.0%) (Fig. 2(c)). Northwest China had a lower atmospheric pressure (854.3 ± 60.3 hPa) compared to East China (1007.4 ± 13.6 hPa) (Fig. 2(d)).

### Correlation between meteorological factors and PM_2.5_

The spearman correlation between meteorological factors and PM_2.5_ is presented in Table S1 in Supplemental Methods. PM_2.5_ was negatively correlated with relative humidity (r = −0.34, P < 0.05). Temperature, atmospheric pressure, and PM_2.5_ were significantly correlated with each other (r = 0.24–0.59, P < 0.05). Atmospheric pressure and relative humidity had a weak positive correlation (r = 0.03, P < 0.05). Since there was no high correlation (|r| > 0.7) among the variables, all variables were included for the following analysis.

### Effects of PM_2.5_ exposure and meteorological factors on AMD

Table 2 shows the odds ratio (OR) with 95% CI for AMD related to increase in PM_2.5_ levels, temperature, relative humidity, and atmospheric pressure, respectively, treated as quartiles. Positive associations with AMD were observed in the adjusted models for the third and fourth quartile of PM_2.5_ with OR (95% CI) of 1.360 (1.267,1.407) and 1.460 (1.321,1.595), respectively. A significant positive association was found between AMD and temperature, relative humidity. The ORs (95% CI) for the second and third quartiles of temperature were 1.972 (1.518,2.562) and 1.341 (1.008,1.785), respectively. Atmospheric pressure was negatively associated with AMD. The ORs (95% CI) for AMD in relation to PM_2.5_ levels, temperature, relative humidity, and atmospheric pressure, treated as continuous variables, are summarized in Table S2.

**Table 2:** Adjusted OR (95%CI) for AMD with meteorological factors and PM_2.5_ separately, treated as quartiles.

	**OR (95% CI) of prevalence of AMD**
PM_2.5_	
Q1	Reference
Q2	0.961 (0.802,1.152)
Q3	1.360 (1.267,1.407)*
Q4	1.460 (1.321,1.595)*
Temperature	
Q1	Reference
Q2	1.972 (1.518,2.562)*
Q3	1.341 (1.008,1.785)*
Q4	1.231 (0.932,1.625)
Relative humidity	
Q1	Reference
Q2	1.610 (1.235,2.099)*
Q3	1.320 (1.003,1.736)*
Q4	1.704 (1.315,2.208)*
Atmospheric pressure	
Q1	Reference
Q2	0.607 (0.512,0.719)*
Q3	0.389 (0.289,0.524)*
Q4	0.141 (0.085,0.232)*

Adjusted for sex, age, regions, ethnicity, education level, occupation, marital status, personal annual income, smoking status, physical activity time, hypertension, and hyperlipidemia. Q1: first quartile; Q2: second quartile; Q3: third quartile; Q4: fourth quartile. *P < 0.05.

**Fig. 3:**
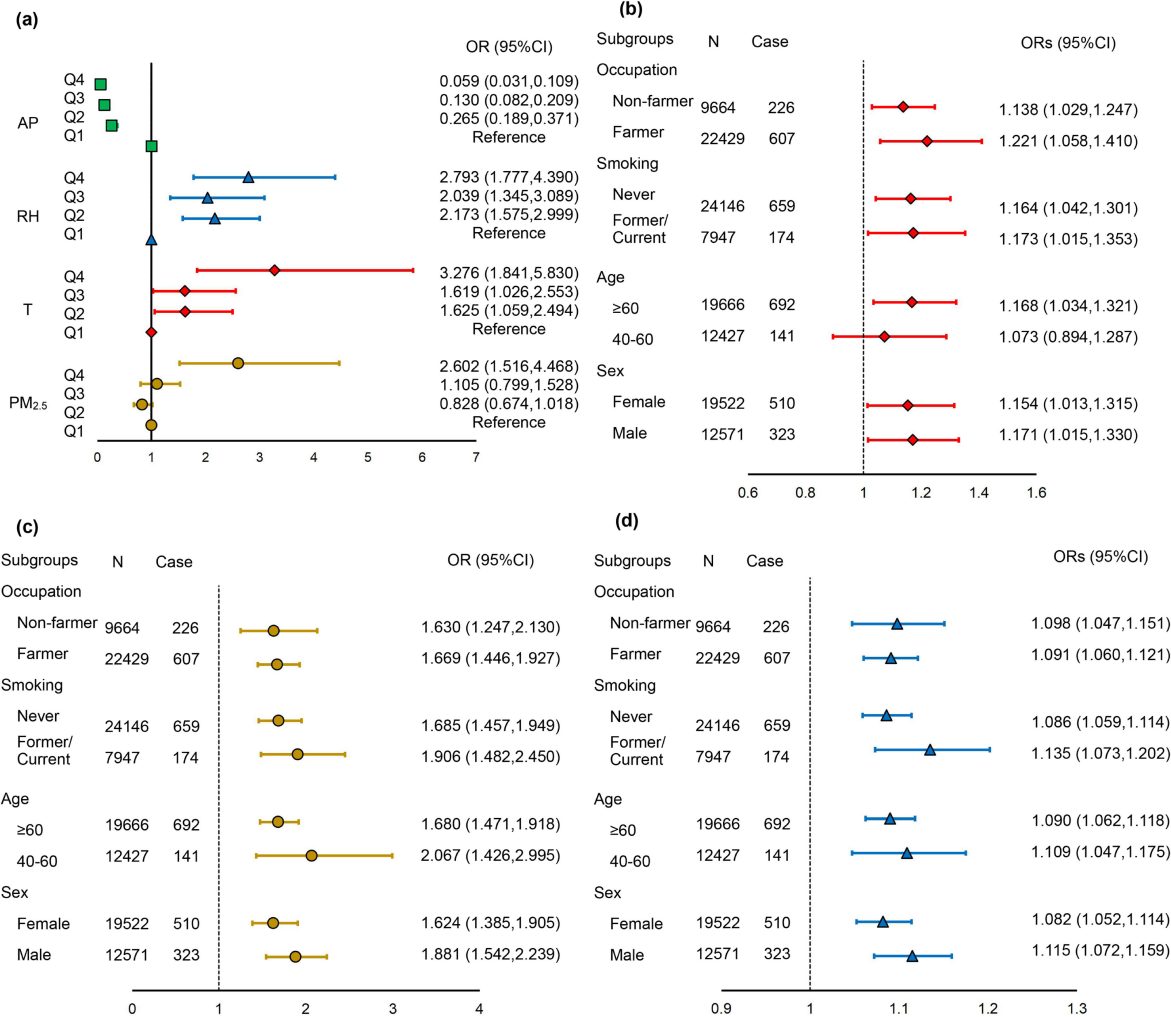
ORs (95%CI) for joint effects and stratified analysis of meteorological factors and PM_2.5_ on AMD. (a): ORs (95%CI) for joint effects of meteorological factors and PM_2.5_ on AMD and PM_2.5_ levels were treated as quartiles, adjusting for age, sex, ethnicity, education level, occupation, smoking status, physical activity time, hypertension, and hyperlipidemia. (b, c, and d): Stratified analysis for joint effects of meteorological factors and PM_2.5_ on AMD, stratified by sex, age, and smoking status. (b): ORs (95%CI) for temperature associated with AMD by subgroups, treated as continuous variables. (c): ORs (95%CI) for PM_2.5_ associated with AMD by subgroups, with each 10 µg/m^3^ increase of PM_2.5_. (d): ORs (95%CI) for relative humidity associated with AMD by subgroups, treated as continuous variables. AP: atmospheric pressure; PM2.5: fine particulate matter; RH: relative humidity; T: temperature; OR: odds ratio; CI: confidence interval.

Joint effects of PM_2.5_ exposure and meteorological factors were analyzed with AMD. As shown in Table S3 in Supplement Methods, as continuous variables, PM_2.5_, temperature, and relative humidity showed positive relationships with AMD, whereas atmospheric pressure exhibited a negative relationship with AMD. In the quartiles variable model, as shown in Fig. 3(a) and Table S4, temperature and relative humidity were significantly associated with AMD. In the combined effect model, the ORs (95% CI) of PM_2.5_ for AMD were 0.828 (0.674,1.018), 1.105 (0.799,1.528), and 2.602 (1.516,4.468) for the second, third, and fourth quartiles, respectively. A positive association was observed between AMD and temperature, with ORs (95% CI) of 1.625 (1.059,2.494) for the second quartile, 1.619 (1.026,2.553) for the third quartile, and 3.276 (1.841,5.830) for the fourth quartile. The effect of relative humidity on AMD showed a similar trend as with temperature, with ORs (95% CI) of 2.173 (1.575,2.999), 2.039 (1.345,3.089), and 2.793 (1.777,4.390), respectively. Atmospheric pressure was negatively associated with AMD when treated as quartiles. The ORs with 95% CIs for effects of atmospheric pressure were 0.265 (0.189,0.371), 0.130 (0.082,0.209), and 0.059 (0.031,0.109) for the second, third, and fourth quartiles, respectively.

### Interactions between PM_2.5_ and meteorological factors on AMD

Interactions between PM_2.5_ and meteorological factors were evaluated by multiplicative and additive interaction terms. On the multiplicative scale, as shown in Table S5 in the Supplementary materials, the OR (95% CI) of the interaction term of cross product between temperature and relative humidity was 0.984 (0.979,0.989), which suggested slight antagonistic effect. The additive interaction of PM_2.5_ and meteorological factors was shown in Table 3, with an estimated RERI and AP of combined PM_2.5_ and atmospheric pressure was 0.864 (0.586,1.141) and 1.180 (0.768,1.592), indicating a significant increase in risk among persons with combined exposure to these factors. The observed RERI suggests that there would be 0.864 relative excess risk due to the additive interaction between PM_2.5_ and atmospheric pressure. The results further show that PM_2.5_ and atmospheric pressure have synergistic effects on AMD. There were no additive interactions between any of the meteorological factors, and the results were shown in Table S6–S8.

**Table 3:** Adjusted OR (95%CI) for AMD by additive interaction analysis of meteorological factors and PM_2.5_ levels.

		**Levels of PM_2.5_ concentration**		**RERI 95%(CI)**	**AP 95%(CI)**	**S 95%(CI)**

		**Low (≤50^th^ percentile)**	**High (>50^th^ percentile)**			
**Temperature**	Low (≤50^th^ percentile)	1.000 (reference)	1.263 (0.877,1.819)	−0.188 (−0.586,0.208)	−0.347 (−1.034,0.341)	1.706 (0.335,8.678)
	High (>50^th^ percentile)	0.469 (0.356,0.619)	0.543 (0.392,0.754)			
**Relative humidity**	Low (≤50^th^ percentile)	1.000 (reference)	1.247 (0.924,1.683)	−0.441 (−0.920,0.037)	−0.397 (−0.831,0.037)	0.201 (0.013,3.083)
	High (>50^th^ percentile)	1.305 (1.004,1.696)	1.111 (0.792,1.559)			
**Atmospheric pressure**	Low (≤50^th^ percentile)	1.000 (reference)	0.685 (0.505,0.931)	0.864 (0.586,1.141)*	1.180 (0.768,1.592)*	0.236 (0.073,0.0.756)
	High (>50^th^ percentile)	0.182 (0.094,0.352)	0.732 (0.457,1.173)			

Adjusted for sex, age, regions, ethnicity, education level, occupation, marital status, personal annual income, smoking status, physical activity time, hypertension, and hyperlipidemia. *P < 0.05.

### Subgroup analyses

Figure 3 (b, c, and d) and Table S9 represent joint effects of PM_2.5_ and meteorological factors on AMD, stratified by sex, age, smoke status, and occupation. The association of temperature and AMD varied by age, as shown in Fig. 3(b). The associations generally were significant in subjects aged 60 or older, with an OR of 1.168 (1.034,1.321). No effect modification by sex, smoking status, or occupation was observed. For PM_2.5_ and relative humidity, the associations were significantly positive with AMD in each stratum. For atmospheric pressure, the associations were significantly negative with AMD in each stratum.

## Discussion

In this national study, the joint effects of meteorological factors and PM_2.5_ on AMD were investigated in rural populations in China. PM_2.5_ levels, temperature, and relative humidity had significant positive associations with AMD, both in independent and combined effect models. Meanwhile, atmospheric pressure was negatively associated with AMD. PM_2.5_ and atmospheric pressure acted synergistically on AMD, as revealed through interaction analysis. Thus, this study presents detailed data revealing both joint and synergistic effects of meteorological factors and PM_2.5_ on AMD. These results warrant attention in prospective cohort and mechanistic studies to further elucidate the relationship between meteorological factors, air pollution, and degenerative eye disease.

The association of PM_2.5_ with AMD in the present investigation was consistent with other studies, such as the UK Biobank study [15], the Canadian Longitudinal Study [16], and a study in Taiwan [14]. The potential mechanisms underlying the effects of air pollution on AMD involve three aspects. First, PM_2.5_ can impair microvascular function. Dysfunction of the microcirculation can lead to age-related diseases, including stroke and AMD, and insufficient microcirculation can promote oxidative stress and inflammatory processes [14, 33]. Second, PM_2.5_ is linked to poor retinal structure, which may lead to AMD [15, 17]. Third, PM_2.5_ can cause neurodegenerative disease, a category that includes AMD [13].

The mechanisms underlying the effects of meteorological factors on AMD remain unclear. However, the influence of meteorological factors on the microvasculature and microcirculation remains one of the most likely mechanisms. In addition, changing weather conditions can indirectly alter eye exposure to air pollutants and allergens [34]. Moreover, it is possible that colder temperatures delay the deterioration of retinal adhesion [35]. It has been proposed that temperature stress affects psychophysiological functions, such as by interfering with cortisol levels in the systemic circulation and thereby increasing intraocular pressure (IOP). IOP can affect blood vessels in the retina and choroid [36]. High IOP compresses the retinal and choroidal blood vessels, resulting in insufficient blood supply to the retinal choroid and ischemia.

The current study also found that AMD is negatively associated with atmospheric pressure. While studies have shown that lower atmospheric pressure may be related to higher IOP, the relationship between AMD and atmospheric pressure remains largely unexplored. Low atmospheric pressure reduces oxygen saturation in the retinal artery, while elevated IOP may further affect oxygen supply to the retina [37, 38]. Further studies are recommended to confirm the negative correlation between AMD and atmospheric pressure.

The interactions of PM_2.5_ and meteorological factors were evaluated by multiplicative and additive interaction terms. The results of this study showed that temperature and relative humidity had a weakly antagonistic effect on AMD. Few studies have explored the effect of relative humidity on temperature-disease associations. Zeng et al. [39] showed that the combination of low temperature and high humidity had the greatest impact on the burden of cardiovascular disease mortality, thus suggesting that a low temperature pose a greater risk of disease at high humidity. At higher temperatures, transpiration of water and soil increases, which leads to a decrease in relative humidity. As a result, it is probable that temperature and relative humidity have an antagonistic effect on AMD, and more research into the underlying mechanisms is required. PM_2.5_ and atmospheric pressure acted synergistic effect on AMD. Spatially, atmospheric pressure played a key role in the distribution of PM_2.5_ concentration, especially in spring and summer [40]. Under low atmospheric pressure, the suspended matter in the atmosphere is difficult to diffuse to the upper air. Air temperature also is known to affect the distribution and concentration of suspended particles, raising the possibility that atmospheric pressure and temperature have combined and possibly synergistic effects on human disease. In this study, however, no significant synergistic effect on AMD was observed between temperature and air pollution. Compared to the independent model, the OR (95% CI) for both temperature and PM_2.5_ increased in the joint effect model.

The results of the stratified analysis also suggest that the association of temperature with AMD varies by age, with effects generally greater in subjects over age 60. A possible reason for this association is that, compared to young persons, older individuals tend to be more sensitive to environmental factors, especially temperature, which can lead to insufficient microcirculation. Decreased microcirculation promotes oxidative stress and inflammatory processes, both of which are risk factors for AMD [41].

This study has some specific strengths. First, it involved a nationwide rural population study with data on 36,081 participants, aged 40 or older, in 10 provinces in China. An established satellite-based spatiotemporal model was used to estimate PM_2.5_ concentrations at 1-km spatial resolution, and a linear interpolate method was used to obtain the meteorological elements of the studied sample points. The sample points were processed to daily average results according to geocoded home addresses, which ensured the relative reliability of the exposure assessment. Several limitations should be noted, however. First, REG-China is a nationwide cross-sectional survey of glaucoma, where causality cannot be determined. Second, the meteorological data was accurate to the home address of each respondent, but the scope of each individual’s activity extends beyond the home address. In addition, individuals in modern society generally spend more time indoors than outdoors, and the meteorological data used are environmental exposures rather than individual exposures, which can lead to measurement errors. Furthermore, it is suggested that more measures against ambient air pollution and global warming should be introduced and implemented.

## Conclusions

In conclusion, the results of the present study provide initial evidence for combined effects of PM_2.5_, temperature, relative humidity, and atmospheric pressure on AMD. The findings provide novel insight into factors that contribute to AMD, which may ultimately help improve AMD prevention and control strategies.

## Abbreviations

AMD: age-related macular degeneration
AOD: aerosol optical depth
AP: atmospheric pressure
CI: confidence interval
ECMWF: European Centre for Medium-Range Weather Forecasts
MODIS: Moderate Resolution Imaging Spectrometer
MAIAC: Multi-Angle Implementation of Atmospheric Correction
NASA: National Aeronautics and Space Administration
OR: odds ratio
OCT: optical coherence tomography
PM: particulate matter
PM_2.5_: fine particulate matter
RH: relative humidity
T: temperature

## Supplementary information

**Additional file 1:** **Table S1:** Correlation coefficient matrix (spearman) of air pollutants and meteorological variables. **Table S2:** Adjusted OR (95%CI) for AMD with meteorological factors and PM_2.5_ separately, treated as continuous variables. **Table S3:** Adjusted OR (95%CI) for joint effects of PM_2.5_ and meteorological factors on AMD (continuous variables). **Table S4:** Adjusted OR (95%CI) for joint effects of PM_2.5_ and meteorological factors on AMD (quartiles). **Table S5:** Adjusted OR (95%CI) for AMD by multiplicative interaction analysis of meteorological factors and PM_2.5_ levels. **Table S6:** Adjusted OR (95%CI) for AMD by additive interaction analysis of temperature and relative humidity. **Table S7:** Adjusted OR (95%CI) for AMD by additive interaction analysis of temperature and atmospheric pressure. **Table S8:** Adjusted OR (95%CI) for AMD by additive interaction analysis of relative humidity and atmospheric pressure. **Table S9:** Adjusted OR (95%CI) for joint effects of PM_2.5_ and meteorological factors on AMD by subgroups. **Fig. S1.** Study areas and locations of twenty-two sites. **Supplemental Methods:** Study population and exposure assessment for PM_2.5_.

## Declarations

### Availability of data and materials

Data will be made available on request.
