# Supplementary material for: Joint effects of meteorological factors and PM2.5 on age-related macular degeneration: a national cross-sectional study in China
Source: Environ Health Prev Med. 2023 Jan 11;28:3. doi: 10.1265/ehpm.22-00237 (PMC9845061; doi:10.1265/ehpm.22-00237)
Supplement: Supplementary file 1 — Additional file 1: Table S1: Correlation coefficient matrix (spearman) of air pollutants and meteorological variables. Table S2: Adjusted OR (95%CI) for AMD with meteorological factors and PM2.5 separately, treated as continuous variables. Table S3: Adjusted OR (95%CI) for joint effects of PM2.5 and meteorological factors on AMD (continuous variables). Table S4: Adjusted OR (95%CI) for joint effects of PM2.5 and meteorological factors on AMD (quartiles). Table S5: Adjusted OR (95%CI) for AMD by multiplicative interaction analysis of meteorological factors and PM2.5 levels. Table S6: Adjusted OR (95%CI) for AMD by additive interaction analysis of temperature and relative humidity. Table S7: Adjusted OR (95%CI) for AMD by additive interaction analysis of temperature and atmospheric pressure. Table S8: Adjusted OR (95%CI) for AMD by additive interaction analysis of relative humidity and atmospheric pressure. Table S9: Adjusted OR (95%CI) for joint effects of PM2.5 and meteorological factors on AMD by subgroups. Fig. S1. Study areas and locations of twenty-two sites. Supplemental Methods: Study population and exposure assessment for PM2.5. [file ehpm-28-003-s001.docx]

**Supplemental Materials**

**Title**

Joint effects of meteorological factors and PM_2.5_ on age-related macular degeneration: a national cross-sectional study in China.

**Authors**

Jiayu He, Yuanyuan Liu, Ai Zhang, Qianfeng Liu, Xueli Yang, Naixiu Sun, Baoqun Yao, Fengchao Liang, Xiaochang Yan, Yang Liu, Hongjun Mao, Xi Chen, Nai-jun Tang, Hua Yan.

**Table of Contents**

**Table S1:** Correlation coefficient matrix (spearman) of air pollutants and meteorological variables.  **(Page 2)**

**Table S2:** Adjusted OR (95%CI) for AMD with meteorological factors and PM_2.5_ separately, treated as continuous variables.  **(Page 3)**

**Table S3:** Adjusted OR (95%CI) for joint effects of PM_2.5_ and meteorological factors on AMD (continuous variables). **(Page 4)**

**Table S4:** Adjusted OR (95%CI) for joint effects of PM_2.5_ and meteorological factors on AMD (quartiles).  **(Page 5)**

**Table S5:** Adjusted OR (95%CI) for AMD by multiplicative interaction analysis of meteorological factors and PM_2.5_ levels. **(Page 6)**

**Table S6:** Adjusted OR (95%CI) for AMD by additive interaction analysis of temperature and relative humidity. **(Page 7)**

**Table S7:** Adjusted OR (95%CI) for AMD by additive interaction analysis of temperature and atmospheric pressure. **(Page 8)**

**Table S8:** Adjusted OR (95%CI) for AMD by additive interaction analysis of relative humidity and atmospheric pressure. **(Page 9)**

**Table S9:** Adjusted OR (95%CI) for joint effects of PM_2.5_ and meteorological factors on AMD by subgroups.  **(Page 10)**

**Fig. S1.** Study areas and locations of twenty-two sites.  **(Page 11)**

**Supplemental Methods:**

Study population and exposure assessment for PM_2.5_.  **(Page 12)**

**Table S1: Correlation coefficient matrix (spearman) of air pollutants and meteorological variables**

|  | PM2.5 | Temperature | RH | AP |
| --- | --- | --- | --- | --- |
| PM2.5 | - | 0.24* | -0.34* | 0.59* |
| Temperature |  | - | 0.58* | 0.40* |
| RH |  |  | - | 0.03* |
| AP |  |  |  | - |

*P < 0.05

**Table S2: Adjusted OR (95%CI) for AMD with meteorological factors and PM_2.5_ separately, treated as continuous variables.**

|  | **Crude model** | **Adjusted model** |
| --- | --- | --- |
| **PM_2.5_** | 1.271 (1.233,1.511) * | 1.192 (1.125,1.365) * |
| **temperature** | 1.042 (1.023,1.061) * | 1.015 (1.002,1.033) * |
| **relative humidity** | 1.076 (1.066,1.087) * | 1.045 (1.032,1.059) * |
| **atmospheric pressure** | 0.993 (0.992,0.994) * | 0.995 (0.994,0.997) * |

Crude model: adjusted no factors associated with AMD and PM_2.5_, meteorological factors

Adjusted Model: adjusted for sex, age, regions, ethnicity, education level, occupation, marital status, personal annual income, smoking status, physical activity time, hypertension, and hyperlipidemia.

* P < 0.05.

**Table S3: Adjusted OR (95%CI) for joint effects of PM_2.5_ and meteorological factors on AMD (continuous variables).**

|  | **OR (95% CI) of prevalence of AMD** |
| --- | --- |
| **PM_2.5_** | 1.706 (1.508,1.930) * |
| **Temperature** | 1.143 (1.035,1.262) * |
| **Relative humidity** | 1.094 (1.069,1.119) * |
| **Atmospheric pressure** | 0.976 (0.970,0.982) * |

Adjusted for sex, age, regions, ethnicity, education level, occupation, marital status, personal annual income, smoking status, physical activity time, hypertension, and hyperlipidemia.

OR (95% CI) for AMD associated with each 10 μg/m 3 increase in PM_2.5_.

OR (95% CI) for AMD associated with each 1℃ increase in Temperature.

OR (95% CI) for AMD associated with each 1% increase in Relative humidity.

OR (95% CI) for AMD associated with each 100Pa increase in Atmospheric pressure.

* P < 0.05.

**Table S4: Adjusted OR (95%CI) for joint effects of PM2.5 and meteorological factors on AMD (quartiles).**

|  | **OR (95% CI) of prevalence of AMD** |
| --- | --- |
| PM_2.5_ |  |
| Q1 | Reference |
| Q2 | 0.828 (0.674,1.018) |
| Q3 | 1.105 (0.799,1.528) |
| Q4 | 2.602 (1.516,4.468) * |
| Temperature |  |
| Q1 | Reference |
| Q2 | 1.625 (1.059,2.494) * |
| Q3 | 1.619 (1.026,2.553) * |
| Q4 | 3.276 (1.841,5.830) * |
| Relative humidity |  |
| Q1 | Reference |
| Q2 | 2.173 (1.575,2.999) * |
| Q3 | 2.039 (1.345,3.089) * |
| Q4 | 2.793 (1.777,4.390) * |
| Atmospheric pressure |  |
| Q1 | Reference |
| Q2 | 0.265 (0.189,0.371) * |
| Q3 | 0.130 (0.082,0.209) * |
| Q4 | 0.059 (0.031,0.109) * |

Adjusted for sex, age, regions, ethnicity, education level, occupation, marital status, personal annual income, smoking status, physical activity time, hypertension, and hyperlipidemia. Q1: first quartile; Q2: second quartile; Q3: third quartile; Q4: fourth quartile. * P < 0.05.

**Table S5: Adjusted OR (95%CI) for AMD by multiplicative interaction analysis of meteorological factors and PM2.5 levels.**

|  | **OR (95% CI) of AMD prevalence** | ***p*-value for the interaction** |
| --- | --- | --- |
| **PM_2.5_** | 1.000 (0.997,1.003) | 0.984 |
| **Temperature** |  |  |
|  |  |  |
| **PM_2.5_** | 0.992 (0.982,1.002) | 0.121 |
| **Relative humidity** |  |  |
|  |  |  |
| **Temperature** | 0.984 (0.979,0.989) | 0.005 |
| **Relative humidity** |  |  |
|  |  |  |
| **PM_2.5_** | 0.998 (0.997,1.000) | 0.180 |
| **Atmospheric pressure** |  |  |
|  |  |  |
| **Temperature** | 1.000 (1.000,1.000) | 0.800 |
| **Atmospheric pressure** |  |  |
|  |  |  |
| **Relative humidity** | 1.000 (0.999,1.001) | 0.100 |
| **Atmospheric pressure** |  |  |

Adjusted for sex, age, regions, ethnicity, education level, occupation, marital status, personal annual income, smoking status, physical activity time, hypertension, and hyperlipidemia.

**Table S6: Adjusted OR (95%CI) for AMD by additive interaction analysis of temperature and relative humidity.**

|  |  | **Temperature** | | **RERI 95%(CI)** | **AP 95%(CI)** | **S 95%(CI)** |
| --- | --- | --- | --- | --- | --- | --- |
|  |  | Low (≤50^th^ percentile) | High (>50^th^ percentile) |  |  |  |
| **Relative humidity** | Low (≤50^th^ percentile) | 1.000 (reference) | 0.831 (0.619,1.115) | -0.261 (-0.628,0.104) | -0.287 (-0.677,0.103) | 0.103  (3.4×10^-15^,3.1×10^12^) |
|  | High (>50^th^ percentile) | 1.342 (1.060,1.699) | 0.912 (0.732,1.134) |  |  |  |

Adjusted for sex, age, regions, ethnicity, education level, occupation, marital status, personal annual income, smoking status, physical activity time, hypertension, and hyperlipidemia.

**Table S7: Adjusted OR (95%CI) for AMD by additive interaction analysis of temperature and atmospheric pressure.**

|  |  | **Temperature** | | **RERI 95%(CI)** | **AP 95%(CI)** | **S 95%(CI)** |
| --- | --- | --- | --- | --- | --- | --- |
|  |  | Low (≤50^th^ percentile) | High (>50^th^ percentile) |  |  |  |
| **Atmospheric pressure** | Low (≤50^th^ percentile) | 1.000 (reference) | 0.827 (0.693,0.987) | 0.061 (-0.157,0.279) | 0.139 (-0.357,0.635) | 0.901 (0.632,1.284) |
|  | High (>50^th^ percentile) | 0.551 (0.383,0.794) | 0.440 (0.318,0.609) |  |  |  |

Adjusted for sex, age, regions, ethnicity, education level, occupation, marital status, personal annual income, smoking status, physical activity time, hypertension, and hyperlipidemia.

**Table S8: Adjusted OR (95%CI) for AMD by additive interaction analysis of relative humidity and atmospheric pressure.**

|  |  | **Relative humidity** | | **RERI 95%(CI)** | **AP 95%(CI)** | **S 95%(CI)** |
| --- | --- | --- | --- | --- | --- | --- |
|  |  | Low (≤50^th^ percentile) | High (>50^th^ percentile) |  |  |  |
| **Atmospheric pressure** | Low (≤50^th^ percentile) | 1.000 (reference) | 1.374 (1.297,1.611) | 0.105 (-0.173,0.384) | 0.755 (-1.492,3.002) | 0.890 (0.670,1.183) |
|  | High (>50^th^ percentile) | 0.659 (0.463,0.938) | 1.139 (1.084,1.230) |  |  |  |

Adjusted for sex, age, regions, ethnicity, education level, occupation, marital status, personal annual income, smoking status, physical activity time, hypertension, and hyperlipidemia.

**Table S9: Adjusted OR (95%CI) for joint effects of PM2.5 and meteorological factors on AMD by subgroups.**

| Characteristic | **OR (95% CI) of prevalence of AMD** | | | |
| --- | --- | --- | --- | --- |
|  | PM_2.5_ | Temperature | Relative humidity | Atmospheric pressure |
| Sex |  |  |  |  |
| male | 1.881 (1.542,2.239) * | 1.171 (1.015,1.330) * | 1.115 (1.072,1.159) * | 0.974 (0.965,0.984) * |
| female | 1.624 (1.385,1.905) * | 1.154 (1.013,1.315) * | 1.082 (1.052,1.114) * | 0.976 (0.968,0.984) * |
| Age |  |  |  |  |
| 40-60 | 2.067 (1.426,2.995) * | 1.073 (0.894,1.287) | 1.109 (1.047,1.175) * | 0.976 (0.965,0.987) * |
| ≥60 | 1.680 (1.471,1.918) * | 1.168 (1.034,1.321) * | 1.090 (1.062,1.118) * | 0.975 (0.967,0.983) * |
| Smoke |  |  |  |  |
| Former/Current | 1.906 (1.482,2.450) * | 1.173 (1.015,1.353) * | 1.135 (1.073,1.202) * | 0.980 (0.966,0.994) * |
| Never | 1.685 (1.457,1.949) * | 1.164 (1.042,1.301) * | 1.086 (1.059,1.114) * | 0.975 (0.968,0.982) * |
| Occupation |  |  |  |  |
| Farmer | 1.669 (1.446,1.927) * | 1.221 (1.058,1.410) * | 1.091 (1.060,1.121) * | 0.971 (0.962,0.980) * |
| Non-farmer | 1.630 (1.247,2.130) * | 1.138 (1.029,1.247) * | 1.098 (1.047,1.151) * | 0.983 (0.974,0.992) * |

* P < 0.05


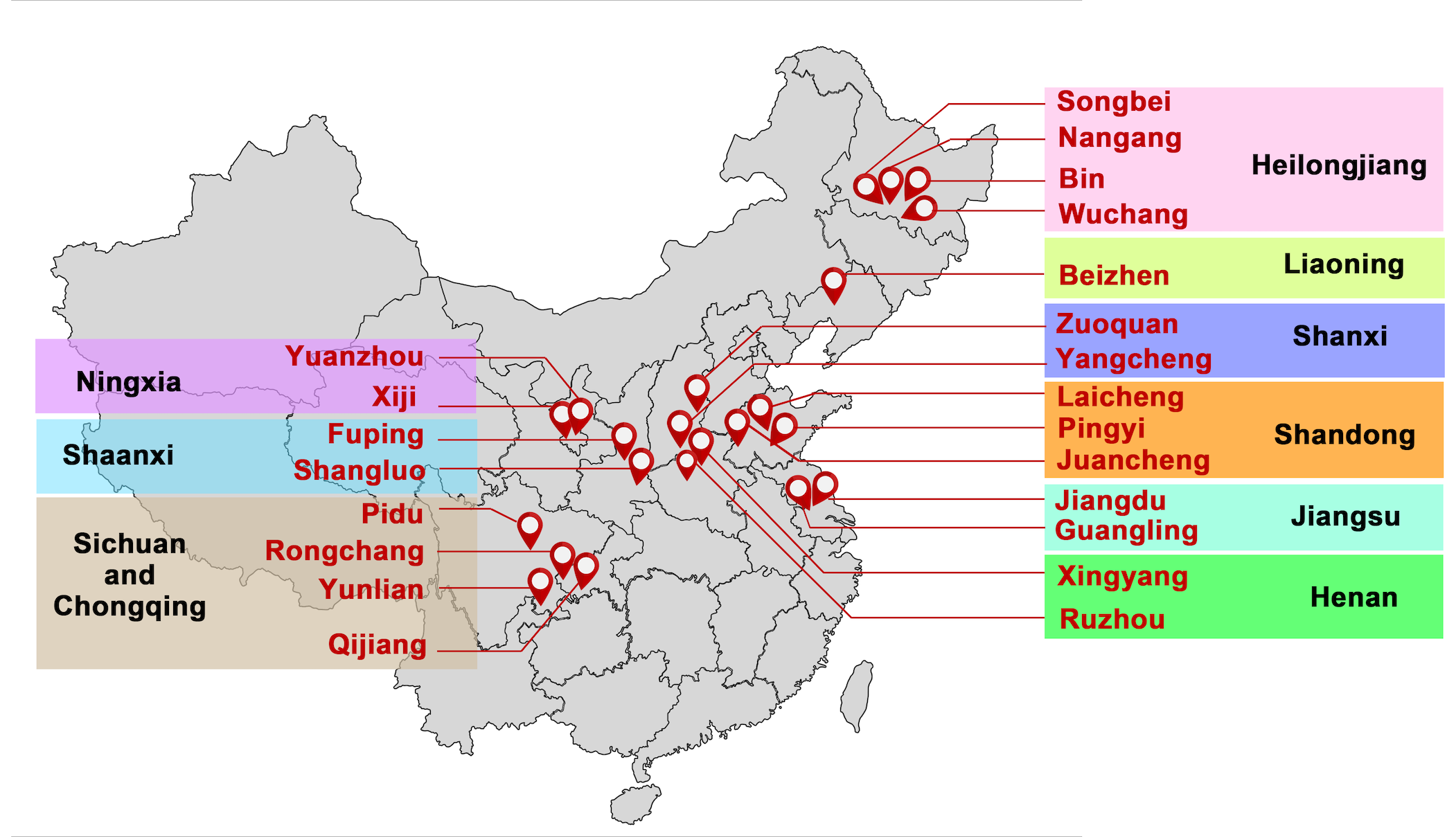


**Fig.S1.** Study areas and locations of twenty-two sites. We identified twenty-two sampling sites in ten provinces by using Study areas and locations of the six regions in China. The Northeast region includes Heilongjiang and Liaoning, the North region includes Shanxi, the East region includes Shandong and Jiangsu, the South-central region includes Henan, the Northwest region includes Ningxia and Shaanxi, and the Southwest region includes Sichuan and Chongqing.

**Supplemental Methods:**

**Study population**

We used a multistage stratified cluster sampling procedure to enroll a nationally representative sample population, aged 6 years or older. A flowchart of the study’s disposition is presented in Figure 2. The study protocol was approved by the Ethics Review Committee of Tianjin Medical University and other participating institutes. Written informed consent was obtained from all participants. Shandong and Jiangsu Provinces were sampled from East China, Shanxi Province was sampled from North China, Heilongjiang and Liaoning Provinces were sampled from Northeast China, Ningxia Hui Autonomous Region, and Shaanxi Province were sampled from Northwest China, Henan Province was sampled from South Central China, and Sichuan Province and Chongqing Municipality were sampled from Southwest China. We used a multistage stratified cluster sampling procedure to enroll a nationally representative sample of populations. It was stratified by region and province, and counties and townships were further selected for each province. The population aged 6 years and above in the sampled districts was investigated and screened. All participants were contacted telephonically and agreed to visit the local community hospital for examination. All organizations and contacts were examined with the assistance of the local community (neighborhood committee) staff. The local community staff understood the household registration and residence information of the community and more frequently came in contact with residents in their daily work. The organization and contact of community workers can enhance the compliance of the population. Standardized training was conducted for all participating clinicians in the early stages of project implementation, and training manuals were developed. The calibration of inspection instruments in each area was also standardized.

**Exposure assessment for PM_2.5_**

First, a daily simple linear regression was performed to impute the missingness for AOD. The regression coefficients were used to estimate the missing Aqua or Terra AOD when only one of them was present (Jinnagara Puttaswamy et al. 2014), and then daily average AOD in each grid cell was used. Second, multiple imputation with an additive model was performed to fill in the remaining missingness, and the predictors included MODIS cloud fraction, humidity in the boundary layer, temperature, elevation, albedo, total column water, and the AOD from modern-era retrospective analysis for research and applications (Randles et al. 2017).

Jinnagara Puttaswamy, S.; Nguyen, H.M.; Braverman, A.; Hu, X.; Liu, Y. Statistical data fusion of multi-sensor AOD over the Continental United States. Geocarto International 2014;29:48-64

Randles, C.A.; Da Silva, A.M.; Buchard, V.; Colarco, P.R.; Darmenov, A.; Govindaraju, R.; Smirnov, A.; Holben, B.; Ferrare, R.; Hair, J.; Shinozuka, Y.; Flynn, C.J. The MERRA-2 Aerosol Reanalysis, 1980 - onward, Part I: System Description and Data Assimilation Evaluation. J Clim 2017;30:6823-6850
